# Ureteral Endometriosis Presenting As Left Ureteral Obstruction: A Case Report

**DOI:** 10.7759/cureus.29288

**Published:** 2022-09-18

**Authors:** Wassim Alaoui Mhammedi, Abdelghani Ouraghi, Mohamed Irzi, Anouar El Moudane, Mohamed Mokhtari, Ali Barki

**Affiliations:** 1 Urology, Mohammed VI University Hospital, Mohammed First University, Oujda, MAR; 2 Urology, Mohammed IV University Medical Center, Oujda, MAR; 3 Urology, Mohamed VI University Hospital, Oujda, MAR; 4 Service Urologie, Chu Mohamed Vi Oujda, Oujda, MAR

**Keywords:** ureterectomy, endometriosis, ureteral endometriosis, ureteric obstruction, urinary obstruction

## Abstract

Ureteral endometriosis is a very rare but serious form of infiltrating endometriosis since the risk of urinary tract obstruction and secondary loss of renal function exists. Although not always possible, the clinical and radiologic assessment may help in obtaining a preoperative diagnosis.

We report the case of a 42-year-old woman with left ureteral endometriosis, revealed by left flank pain. Imaging revealed left obstructive uropathy with an endometriotic cyst of the left ovary and a spiculated lesion of the left parametrium. She underwent laparotomy, resection of the diseased ureter with primary re-anastomosis, resection of a left parametrial lesion and an endometriotic left ovarian cystectomy. The pathological assessment confirmed the diagnosis of ureteral endometriosis. Follow-up of the patient showed complete resolution with a stable, normal kidney function.

In conclusion, ureteral endometriosis involvement is infrequent but should be included in the differential diagnosis in a premenopausal woman with ureteral obstruction of unknown cause. An early diagnosis and obstruction relief are critical to a successful outcome.

## Introduction

Endometriosis is defined by the ectopic presence of functional endometrial glands and/or stroma outside the uterus [1]. Many theories exist to explain this phenomenon, the most accepted one being retrograde menstruation entering the peritoneal cavity through the fallopian tubes. Depending on its location, endometriosis can be divided into 3 categories: superficial (when located in the peritoneum), ovarian and deep infiltrating endometriosis (DIE) [2,3].

This later form is defined by the presence of endometrial implants penetrating at a level greater than 5mm, beneath the peritoneal surface. This form of endometriosis can involve the uterosacral ligaments, the rectovaginal space, and intraperitoneal organs such as the bowel and urinary tract [2]. In terms of urinary tract involvement, pelvic endometriosis involves the urinary tract in 1% cases. Ureteral endometriosis is present in 10% of all urinary tract cases [4].

We report the case of a 42-year-old woman with left ureteral endometriosis, revealed by left flank pain. Imaging revealed left obstructive uropathy with an endometriotic cyst of the left ovary and a spiculated lesion of the left parametrium. She underwent laparotomy, resection of the diseased ureter with primary re-anastomosis, resection of a left parametrial lesion and an endometriotic left ovarian cystectomy.

## Case presentation

A 42-year-old primi-parous patient presented with a one-month history of progressively worsening non-cyclical left flank pain. The patient had no significant past medical history. She reported no abnormal vaginal discharge or bleeding and no urinary symptoms. Clinical examination revealed left flank and pelvic tenderness. A left pelvic mass was also found during a vaginal examination. No other significant findings were present.

The complete blood count was normal. The kidney function was normal. Urinary tract ultrasound revealed an important left moderate hydronephrosis reaching 15cm in greatest diameter with identification of a heterogenous lesion of the left ureter measuring less than 1cm, next to a ureterovesical junction (UVJ). Ultrasound could also identify the presence of a 5cm left ovarian cystic lesion, suggestive of an endometriotic cyst.

**Figure 1:**
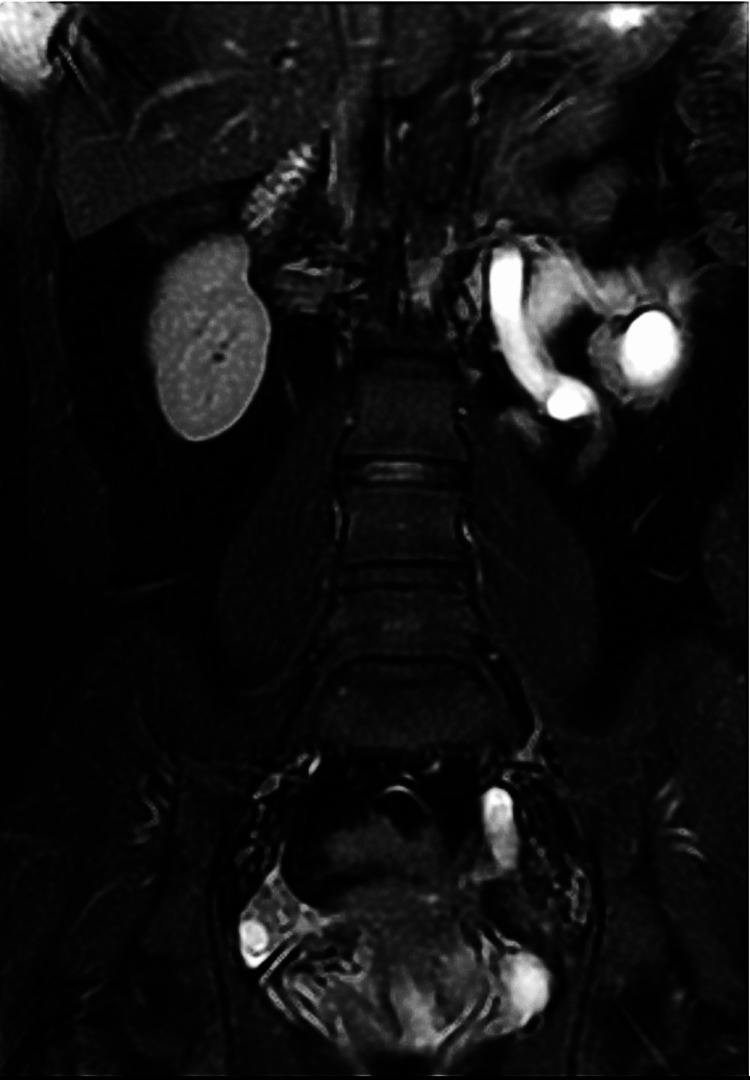
T2 FAT SAT MRI sequence image showing left hydro-uretero-nephrosis secondary to ureteral stenosis. The stenosis is in communication with a spiculated lesion of the left parametrium.

**Figure 2:**
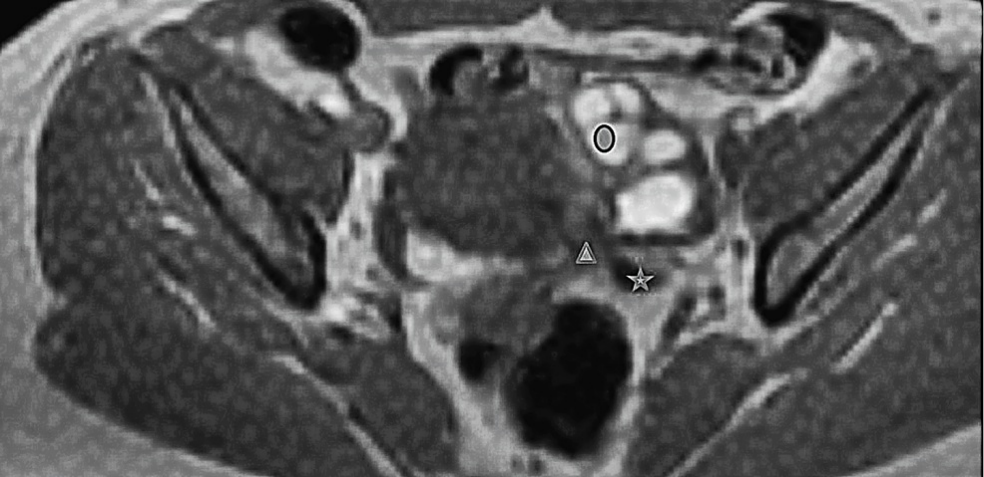
T1 FSE MRI sequence image showing the presence of a left ureteral stenosis (Star), with the presence of a spiculated lesion on the left parametrium (Triangle). A hematosalpinx could also be identified.

Abdominal pelvic Magnetic resonance imaging (MRI) was performed on our patient, in T2 FAT SAT (Fat Saturation) and T1 FSE (Fat Spin Echo) sequences, revealing the presence of left ureteral stenosis in communication with a spiculated lesion of the left parametrium. MRI also identified the presence of a left hematosalpinx and confirmed the endometriotic nature of the left ovarian cyst (Figures 1, 2).

 

Decision was made to surgically remove the ureteral stenosis. Intra-operatively, the presence of a 5cm left ovarian cyst was confirmed. The uterus, the right ovary and tubes showed no lesions. Exploration of the left retro-peritoneal space revealed left hydroureteronephrosis with the presence of a spiculated lesion of the left parametrium that was invading the left ureter. A partial ureterectomy was performed in our patient by excising the diseased ureteral segment with excision of the left parametrial spiculated lesion. An anastomosis of the distal and proximal ends was performed using an interrupted 4-0 polyglactin suture with placement of a ureteral double J stent. A resection of the left ovarian cyst was also performed.

**Figure 3:**
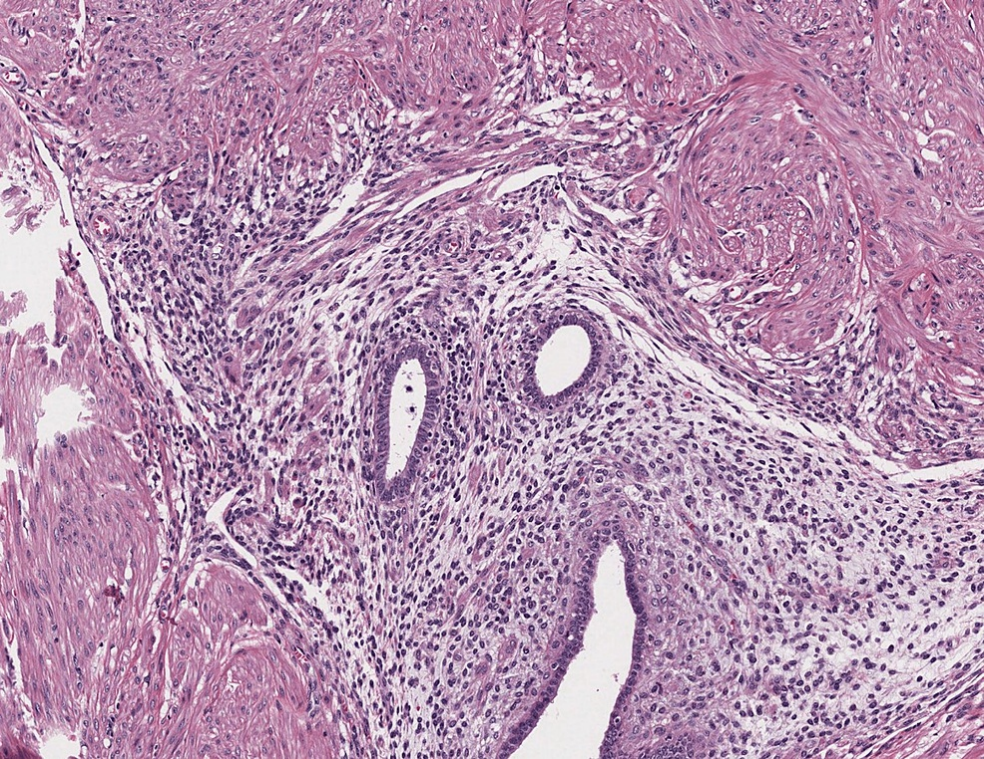
Photomicrograph showing a focus of endometrial glands and stroma within the muscularis propria of the ureteral wall (HE; 200x).

**Figure 4:**
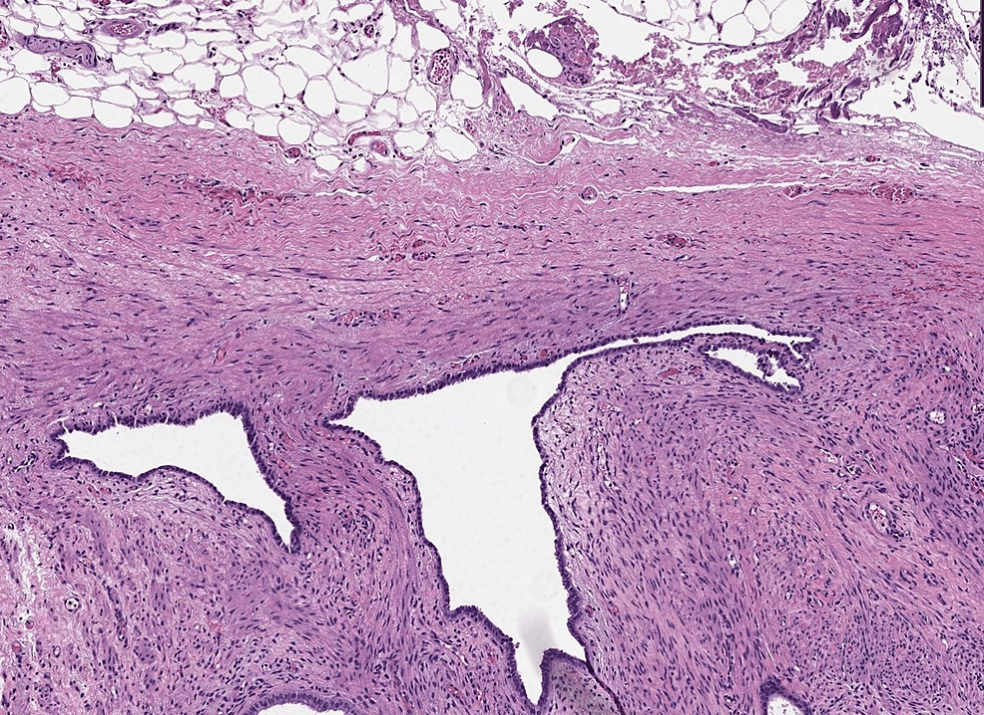
Photomicrograph showing presence of endometrial glands and stroma in the spiculated lesion of the left parametrium (HE; 200x).

**Figure 5:**
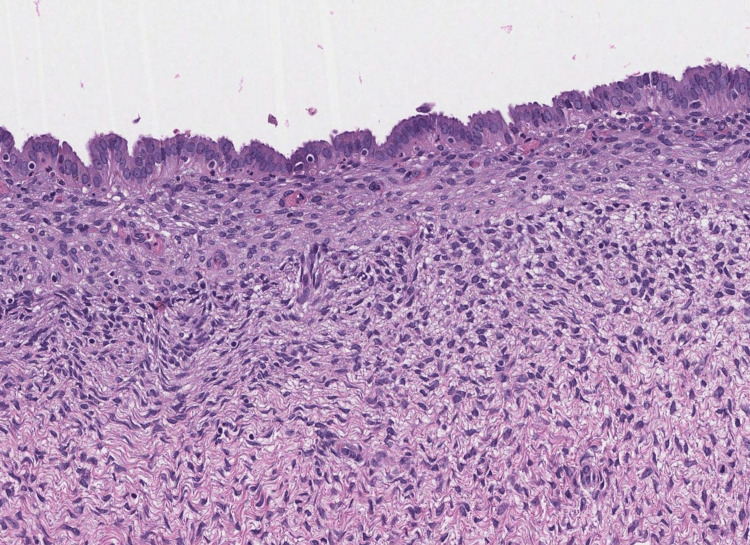
Photomicrograph showing evidence of the endometriotic nature of the left ovarian cyst. The wall is layered by an endometrial epithelium overlaying an endometrial stroma. A regular ovarian stroma can be seen beneath (HE; 200x).

Pathological assessment showed the presence of foci of endometrial glands and stroma on the muscularis propria of the ureteral wall (Figure 3), in the left parametrial spiculated lesion (Figure 4) and on the left ovarian cyst confirming its endometriotic nature (Figure 5).

The double J stent was removed six weeks post-operatively. Follow-up through biological exploration and renal ultrasound revealed total regression of left hydroureteronephrosis with a persistent normal renal function. The patient was put under daily Dienogest 2mg. The evolution of our patient was favorable with no symptom recurrences.

## Discussion

The urinary tract is rarely involved in endometriosis patients. Ureteral endometriosis as in our patient is the second most frequent urinary location of endometriosis (10% of cases), with the urinary bladder being the first one with ratios of 40:5:1:1 for Bladder/ureter/kidney and urethra respectively [5]. Pelvic endometriosis involves the urinary tract in 1% cases [4].

On the pathological level, ureteral endometriosis can be intrinsic when endometrial tissue is in the lamina propria or tunica muscularis of the ureter or extrinsic when endometrial tissue is present in the peri-ureteral tissue [6]. Until now the most accepted theory explaining the occurrence of ureteral endometriosis is through retrograde menstruation [6]. This theory is furthermore supported by the presence of a right/left asymmetry since the sigmoid colon enables establishing a micro-environment around the left adnexa favoring interaction between endometrial cells and peritoneum in this area [7]. A second theory exists sand may link the occurrence of ureteral endometriosis to embryonic remains of the Mullerian duct. [8,9]

A hereditary component should also be present since endometriosis is increased in first-degree relatives of women with this disease [7]. The reported symptoms depend on the extension of endometrial tissue deposits and their location. The leading symptoms are chronic pelvic pain, dysmenorrhea, deep dyspareunia, cyclical intestinal complaints, fatigue, and infertility [10]. In cases of urinary endometriosis, reported symptoms are not specific, as a study conducted by Soriano et al. in 2011 revealed an incidence of 95.5% for dysmenorrhea, 60% for dyspareunia and only 15.9% for urinary symptoms [11]. Other rare reported symptoms in cases of urinary tract endometriosis include hypertension [12] and anuria [13]. In these cases, the risk of renal failure would be as high as 25%-50% [14]. Since symptoms are non-specific, exploration of the urinary tract is suggested in cases of suspected deep infiltrating endometriosis, especially when nodules greater than 3cm are present in the rectovaginal septum [15]. As in our reported case, the non-specific nature of symptoms can be misleading for a preoperative diagnosis of ureteral endometriosis, which is difficult in most cases.

This diagnosis could be suggested preoperatively in only 40% of patients, as shown in one study [15]. In our case, the presence of a left endometriotic ovarian cyst helped to suggest the diagnosis of ureteral endometriosis. Clinical examination is generally poor but can identify rectovaginal large endometriotic nodules, which is highly suggestive of an associated ureteral involvement [13]. An assessment of renal function should be performed to identify any kidney function alterations, hematuria, or signs of malignant disease [14].

Imaging can play a great role in preoperative diagnosis. Renal ultrasound can identify urinary tract obstruction and may also, as in our case, identify the presence of a ureteral lesion [14]. CT scan, which is the most used imaging technique, could help in establishing a preoperative diagnosis by determining the size, the location, the extent of the endometrial focus and the presence of urinary obstruction [14]. Ureteroscopy helps to directly visualize endometrial implant foci on the ureteral wall. These appear as oedematous and irregular nodules with different possible shapes and colors. It also allows biopsies for histological confirmation although negative results do not exclude the diagnosis [15] Ureteroscopy also helps in guiding the therapeutic surgical approach by measuring the distance between the lower endometriotic margins and the ureteral orifice [12].

MRI has since its apparition replaced Intravenous pyelography (IVP) and retrograde pyelography and remains as the best diagnostic tool to assess the disease extension and plan an adequate surgical approach. Sillou et al. in 2015 demonstrated that MRI has a sensitivity as high as 91%, but with a much lower specificity of 59% [16].

The management of cases of ureteral endometriosis is different from one patient to another. Medical treatment can be indicated in early-stage disease but presents an incomplete response and high rates of relapse after cessation of hormonal therapy [17]. Medical treatment is based on progestogens and gonadotropin-releasing hormone agonists [17]. Medical treatment should never delay a surgical treatment of hydronephrosis since a risk of renal function loss exists [18].

The surgical treatment of intrinsic ureteral endometriosis is based on resection and reconstruction of the ureter, with two existing techniques: Ureteral-ureteral anastomosis, indicated in cases of limited disease with a potentially preservable ureter [6]. This technique has a high recurrence rate [19]. The second option is through ureteroneocystostomy, indicated especially in cases of extended disease, when the vesicoureteral junction is involved and when the distal ureteral intact stump is 1cm long or less [20].

Surgical techniques also include nephrectomy, indicated in cases of renal function loss, which should be confirmed by renal scintigraphy that enables estimation of the residual renal function, or in cases where diagnosis surgery seems to be necessary because of malignancy suspicion. As for our case, who presented with a synchronous left ovarian endometriotic cyts, up to 90% of women with ureteral endometriosis had endometriosis in other sites, as demonstrated in a study by Seraccholi et al. [20]. On the prognostic level, favorable outcomes, particularly regarding renal function can be reached when diagnosis and surgery are early, with a long-term follow-up [20].

## Conclusions

Ureteral endometriosis is a rare and challenging diagnosis because of the nonspecific nature of reported symptoms. Radiology, especially MRI and cystoscopy are the gold standard diagnostic tools for a preoperative diagnosis. Surgery remains the best therapeutic option, with existence of medical hormonal therapy, but with less promising results. A greater awareness is needed for early diagnosis and management since they are both key to better outcomes.
